# Activation of the Regulatory T-Cell/Indoleamine 2,3-Dioxygenase Axis Reduces Vascular Inflammation and Atherosclerosis in Hyperlipidemic Mice

**DOI:** 10.3389/fimmu.2018.00950

**Published:** 2018-05-07

**Authors:** Maria J. Forteza, Konstantinos A. Polyzos, Roland Baumgartner, Bianca E. Suur, Marion Mussbacher, Daniel K. Johansson, Andreas Hermansson, Göran K. Hansson, Daniel F. J. Ketelhuth

**Affiliations:** ^1^Cardiovascular Medicine Unit, Center for Molecular Medicine, Department of Medicine, Karolinska Institute, Karolinska University Hospital, Stockholm, Sweden; ^2^Center for Molecular Medicine, Department of Molecular Medicine and Surgery, Karolinska Institute, Stockholm, Sweden; ^3^Department of Vascular Surgery, Karolinska University Hospital, Stockholm, Sweden; ^4^Department of Vascular Biology and Thrombosis Research, Center for Physiology and Pharmacology, Medical University of Vienna, Vienna, Austria

**Keywords:** atherosclerosis, T-cell, regulatory T cell, IDO, tryptophan, kynurenine

## Abstract

T-cell activation is characteristic during the development of atherosclerosis. While overall T-cell responses have been implicated in disease acceleration, regulatory T cells (Tregs) exhibit atheroprotective effects. The expression of the enzyme indoleamine 2,3-dioxygenase-1 (IDO1), which catalyzes the degradation of tryptophan (Trp) along the kynurenine pathway, has been implicated in the induction and expansion of Treg populations. Hence, Tregs can reciprocally promote IDO1 expression in dendritic cells (DCs) *via* reverse signaling mechanisms during antigen presentation. In this study, we hypothesize that triggering the “Treg/IDO axis” in the artery wall is atheroprotective. We show that apolipoprotein B100-pulsed tumor growth factor beta 2-treated tolerogenic DCs promote *de novo* FoxP3^+^ Treg expansion *in vivo*. This local increase in Treg numbers is associated with increased vascular IDO1 expression and a robust reduction in the atherosclerotic burden. Using human primary cell cultures, we show for the first time that IDO1 expression and activity can be regulated by cytotoxic T-lymphocyte associated protein-4, which is a constitutive molecule expressed and secreted by Tregs, in smooth muscle cells, endothelial cells, and macrophages. Altogether, our data suggest that Tregs and IDO1-mediated Trp metabolism can mutually regulate one another in the vessel wall to promote vascular tolerance mechanisms that limit inflammation and atherosclerosis.

## Introduction

Atherosclerosis is a chronic inflammatory disease initiated by the retention and accumulation of low-density lipoprotein (LDL) in the artery wall. Trapped in the intima, the lipid moiety of LDL or apolipoprotein B100 (ApoB100) have been identified as major drivers of this disease, triggering the response of vascular, innate, and adaptive immune cells (1). While overall T-cell responses have been implicated in the aggravation of atherosclerosis, regulatory T cells (Tregs) have been identified as critical for protection against disease (2). Tregs can exert their antiatherogenic effects *via* the local secretion of anti-inflammatory cytokines, such as transforming growth factor beta (TGFβ) and interleukin 10 (IL-10), or by cell-to-cell contact (3).

We have previously shown that the immunomodulation of T-cell responses can reduce vascular inflammation and plaque formation (4–6). Indoleamine 2,3-dioxygenase-1 (IDO1), which is the rate-limiting enzyme catalyzing the production of metabolites in the Kynurenine pathway of tryptophan (Trp) degradation, has been implicated in the regulation of T-cell effector responses and the expansion of Tregs (7–9). The inhibition of Trp metabolism using the IDO inhibitor 1-methyl tryptophan (1-MT) or the genetic ablation of IDO1 in hypercholesterolemic mice results in a substantial increase in vascular inflammation and accelerated plaque formation (10, 11).

Pro-inflammatory signaling pathways, including toll-like receptors, tumor necrosis factor superfamily members, type I and II interferons, and the aryl hydrocarbon receptor, have been implicated in the regulation of IDO expression (12). Nevertheless, Treg signals, such as TGFβ and cytotoxic T-lymphocyte associated protein-4 (CTLA4), have been also shown to influence IDO1 expression in antigen-presenting cells (APCs) in animals and humans (13, 14).

In this study, we hypothesized that the activation of the “Treg/IDO axis” in the vascular wall can modulate atherosclerosis. We show that promoting the expansion of antigen-specific FoxP3^+^ Tregs in the artery wall with an injection of tumor growth factor beta 2 (TGFβ_2_)-treated and ApoB100-pulsed tolerogenic dendritic cells (DCs) leads to increased IDO1 expression and atheroprotection. Indeed, we show that CTLA4 is a major regulator of IDO1 expression and activity in vascular cells and macrophages. Our data reveal novel mechanisms underlying the maintenance of immunohomeostasis in the vascular wall. Thus, the induction of the “Treg/IDO axis” emerges as a promising therapeutic approach for the prevention and treatment of atherosclerotic cardiovascular diseases (CVDs).

## Materials and Methods

### Animals

Human ApoB100-transgenic *Ldlr^−/−^* mice [*HuBL, B6.C57BL/6XSJL-^*Tg(huB100tm)*^*0.129S7-Ldlr*^*tm1Her*^* (15, 16)] were used for the generation of the bone marrow-derived DCs and the atherosclerosis experiments. T cells from C57BL6/J mice were used in the Treg conversion assays.

### Preparation of ApoB100

Low-density lipoprotein (1.019−1.063 g/mL) was isolated from pooled plasma from healthy donors by sequential ultracentrifugation as previously described (17). ApoB100 was isolated by the addition of four parts of methanol, one part of chloroform, and three parts of water to one part of LDL. Then, the mixture was vortexed and centrifuged at 9,000 × *g* for 10 min, which resulted in protein precipitation at the chloroform–methanol–water interphase. Then, ApoB100 was dissolved in sodium dodecyl sulfate, filtered using a PD-10 column (GE Healthcare Life Sciences, Uppsala, Sweden), and purified by high-pressure liquid chromatography using a Superdex200 (GE Healthcare Life Sciences, Uppsala, Sweden) size-exclusion column (0.5 mL/min in Tris-buffered saline, pH 7.6).

### Preparation of Bone Marrow-Derived DCs

The DCs were isolated as previously described (5). Briefly, bone marrow cells from the femur and tibia bones of *HuBL* mouse donors were depleted of red blood cells and cultured at 37°C and 7.5% CO_2_ for 8 days in medium (DMEM, 10% FCS, 50 U/mL penicillin, 50 g/mL streptomycin, 1 mmol/L sodium pyruvate, 2 mmol/L l-glutamine) supplemented with 10 ng/mL IL-4 and 10 ng/mL GM-CSF (PeproTech, NJ, USA). The generated DCs were purified by positive selection using CD11c magnetic cell-sorting kit (Miltenyi Biotec, Bergisch Gladbach, Germany) according to the manufacturer’s instructions.

### *De Novo* Induction of Tregs *In Vitro*

The conversion of Tregs *in vitro* was investigated using CD11c^+^ DCs that were incubated with either 5 ng/mL TGFβ_2_ (R&D Systems, MN, USA) or 10 µg/mL IL-10 (R&D Systems, MN, USA) for 24 h. A group without cytokine treatment was used as a control. After washing, the DCs were cocultured at 37°C and 7.5% CO_2_ for 48 h with CD4^+^CD25^−^ naïve T cells obtained by negative selection (Miltenyi Biotec, Bergisch Gladbach, Germany) from spleens from C57BL6/J mice. The polyclonal conversion of Tregs was induced by stimulation with 1 µg/mL anti-CD3 (R&D Systems, MN, USA) and 2 µg/mL anti-CD28 (R&D Systems, MN, USA). After 48 h, the percentage of CD4^+^CD25^+^FoxP3^+^Ki67^+^ cells was assessed by flow cytometry.

### Treg Generation *In Vivo* by TGFβ_2_ Tolerogenic DCs

We have previously shown that IL-10-generated tolerogenic DCs can induce antigen-specific Treg formation *in vitro* and *in vivo* (5). In this study, we show that TGFβ_2_ has a superior capacity to induce Treg conversion *in vitro*; thus, these DCs were selected for the *in vivo* experiments. The DCs, which were generated as previously described, were incubated in tissue culture dishes with 5 ng/mL TGFβ_2_ (R&D Systems, Minneapolis, MN, USA) with or without 25 µg/mL ApoB100, in serum-free DMEM medium containing insulin, human transferrin, selenous acid (1:100 ITS Premix, Biosciences, Franklin Lakes, NJ, USA), 1 mmol/L sodium pyruvate (Gibco Invitrogen, Carlsbad, CA, USA), 1 mg/mL bovine serum albumin (Sigma-Aldrich, St. Louis, MO, USA), 1 mmol/L non-essential amino acids (Sigma-Aldrich, Stockholm, Sweden), 10 mmol/L HEPES (Gibco Thermo Fisher Scientific, MA, USA), and 50 g/mL gentamicin sulfate (Sigma-Aldrich, St. Louis, MO, USA) at 37°C and 5% CO_2_. After 4 h, 0.1 ng/mL lipopolysaccharide was added, and the cells were incubated for an additional 14 h. Finally, the DCs were washed with DMEM, maintained on ice, and injected into recipient mice within 1 h. Cytokine secretion, including IL-10, IL-12, and TNF, by the DCs was analyzed in the supernatants of cultures by ELISA (R&D Systems, Minneapolis, MN, USA) according to the manufacturer’s instructions.

Eleven-week-old male HuBL mice were injected with 2.5 × 10^5^ DCs that had been loaded or not with ApoB100 and treated with or without TGFβ_2_. Five days after the DC transfer, the mice were fed a Western diet (corn starch, cocoa butter, casein, glucose, sucrose, cellulose flour, minerals, and vitamins; 17% protein, 21% fat, 0.15% cholesterol, 43% carbohydrates, 10% H_2_O, and 3.9% cellulose fibers; R638 Lantmännen, Kimstad, Sweden) for 10 weeks.

### Tissue Processing, Immunostaining, and Lesion Analysis

After sacrifice, blood was collected by cardiac puncture, and vascular perfusion was performed using sterile RNase-free PBS. The abdominal aorta was dissected and snap-frozen for the subsequent RNA isolation. The heart and aortic arch were dissected and preserved for the immunohistochemistry and plaque analyses as previously described (18). *En fac*e lipid accumulation was determined in the thoracic aorta from the immunized mice using Sudan IV staining. The plaque area was calculated as the percentage of the total surface area of the thoracic aorta. The plaque cell markers in sections of aortic roots was evaluated using primary antibodies against vascular cell adhesion molecule 1 (VCAM-1) (all BD Biosciences, Franklin Lakes, NJ, USA); CD68 (AbD Serotec, Kidlington, UK), α-smooth muscle-actin (αSMA) (Abcam, Cambridge, UK); CD31 (Abcam, Cambridge, UK); FoxP3 (eBioscience, Thermo Fisher Scientific, MA, USA); and IDO1 (BioLegend, San Diego, CA, USA) that were applied to acetone-fixed cryosections, and against l-kynurenine (ImmuSmol, Pessac, France) that was applied to paraformaldehyde-fixed cryosections. The detection was performed using an ABC alkaline phosphatase kit (Vector Laboratories, Burlingame, CA, USA) or Envision system (Dako, Copenhagen, Denmark) as previously described (18, 19). Immunofluorescence staining was performed using goat anti-rat IgG (Dylight^®^ 594) and horse anti-rabbit (Dylight^®^ 488) as the secondary antibodies (Vector Laboratories, Burlingame, CA, USA), and nuclei were stained with DAPI (Sigma-Aldrich, MO, USA).

### Plasma Analysis

The plasma cholesterol and triglyceride levels were measured using enzymatic colorimetric assays (Randox Laboratories, Crumlin, UK) according to the manufacturer’s protocol.

### Flow Cytometry Analysis

The characterization of the DC and T-cell phenotypes was performed by flow cytometry (CyAnTM; Dako, Glostrup, Denmark). Primary antibodies against murine CD11c, I-Ab, CD11b, CD205, CD86, Ki67 (all from BD Biosciences, NJ, USA) and FoxP3 (eBioscience, Thermo Fisher Scientific, MA, USA) were used. The results were acquired using FlowJo software (TreeStar software, Ashland, OR, USA).

### Quantitative PCR

RNA was isolated using an RNeasy kit (Qiagen, Hilden, Germany), reverse-transcribed, and amplified by real time-PCR using Assay-On-Demand primers and probes (Applied Biosystems, CA, USA). Hypoxanthine guanidine ribonucleosyl transferase was used as a housekeeping gene. The relative expression was calculated using the formula 2^−ΔΔCt^, where ΔΔCt = ΔCt (sample) − ΔCt (calibrator = average Ct of controls), and ΔCt is the average Ct of the housekeeping gene subtracted from the target gene Ct.

### Human Macrophage Cultures

Peripheral blood was obtained from healthy volunteers at the Blood Central of Karolinska University Hospital, Stockholm, Sweden. Peripheral blood mononuclear cells were isolated using Lymphoprep™ gradient medium (density 1.077 g/ml; Axis-Shield, Oslo, Norway) according to the manufacturer’s instructions. After a 1 h adherence step, the floating cells were discarded. The adherent monocytes were used to generate “M0” macrophages as previously described (20). Briefly, the monocytes were cultured for 6 days in medium [RPMI 1640, 50 U/mL penicillin, 50 g/mL streptomycin (Gibco Invitrogen, Carlsbad, CA, USA), and 10% fetal bovine serum] supplemented with 20 ng/mL M-CSF (R&D Systems, MN, USA). After 24 h of pre-stimulation with human recombinant IFNγ (400 U/mL), the cells were washed three times with PBS and incubated with medium alone, recombinant human CTLA4-Ig (hum/hum) or recombinant human IgG1-Fc isotype control (both from BioXCell, Lebanon, NH, USA) for 24 h. Unstimulated macrophages were used as controls. The supernatants were collected for the determination of IDO activity by HPLC.

### Human Smooth Muscle Cell (SMC) Cultures

Commercial human aortic SMCs (Cascade Biologics, Life Technologies, CA, USA) were maintained in SMC medium (Lonza, Basel, Switzerland). After 24 h of pre-stimulation with human recombinant IFNγ (400 U/mL), the cells were washed three times with PBS and incubated with medium alone, recombinant human CTLA4-Ig (hum/hum) or recombinant human IgG1-Fc isotype control (both from BioXCell, Lebanon, NH, USA) for 24 h. Unstimulated SMCs were used as controls. The supernatants were collected for the determination of IDO activity by HPLC.

### Human Umbilical Vein Endothelial Cell (HUVEC) Cultures

Human umbilical vein endothelial cells were cultured in Medium 199 supplemented with 20% (v/v) fetal bovine serum, 100 U/mL penicillin, 0.1 mg/mL streptomycin, 2 mM l-glutamine, 1 µg/mL heparin (Sigma-Aldrich, St. Louis, MO, USA), and 10 µg/mL endothelial cell growth factor supplement (Sigma-Aldrich, St. Louis, MO, USA). Cells from passage 1 or 4 were used in all experiments. Cells grown to confluence were pretreated with INF-γ (400 U/mL) at 37°C for 24 h. After three washes with PBS, the cells were incubated with medium alone, recombinant human CTLA4-Ig (hum/hum) or recombinant human IgG1-Fc isotype control (both from BioXCell, Lebanon, NH, USA) for an additional 24 h. Unstimulated HUVECs were used as controls. The supernatants were collected for the determination of IDO activity by HPLC.

### IDO Activity Assay

The Kyn to Trp ratio (Kyn/Trp) was used as a surrogate marker of IDO activity. The Trp and Kyn levels in the plasma and tissues were analyzed by isocratic liquid chromatography with ultraviolet detection as previously described (21).

### Arterial IDO Quantification by Western Blotting

The total protein was extracted from frozen aortic root sections as previously described (22). Ten micrograms of extract were separated by SDS–PAGE (4–15%, Bio-Rad Laboratories, CA, USA) and transferred to PVDF membranes (GE Healthcare, Uppsala, Sweden). The membranes were probed for murine IDO using an M-48 antibody (BioLegend, San Diego, CA, USA). An α-tubulin quantification (anti-α-tubulin; Abcam, Cambridge, UK) was performed as a loading control.

### Statistics

The non-parametric Mann–Whitney *U*-test was used for comparisons between two groups. Comparisons among more than two groups were performed using non-parametric Kruskal–Wallis ANOVA, followed by a Dunn’s multiple comparison *post hoc* test. The correlations were calculated using the Spearman’s rank test. The differences were considered significant at *P*-values < 0.05 (two-tailed). All statistical analyses were performed using GraphPad Prism version 6.0f for Mac OS X (GraphPad Software, Inc., CA, USA).

## Results

### TGFβ_2_ Induces a Potent Tolerogenic Phenotype in DCs

**Figure 1:**
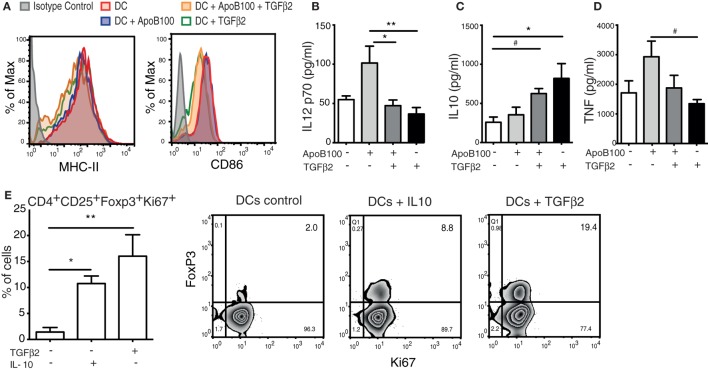
Tumor growth factor beta 2 (TGFβ_2_)-induced tolerogenic dendritic cells (DCs) generate *de novo* regulatory T cells (Tregs) *in vitro*. **(A)** Representative flow cytometry overlaid histograms of MHC-II and CD86 expression in bone marrow-derived DCs incubated with different treatments. **(B–D)** Levels of IL-12 p70, interleukin 10 (IL-10), and TNF from the supernatants of bone marrow DCs incubated with different treatments. The results are pooled data from four independent experiments (duplicate wells). Pooled cells from five mice were used in each experiment. Unstimulated DCs were used as a control. **(E)** Proliferation and *de novo* induction of Tregs (CD4^+^CD25^+^FOXP3^+^KI67^+^ cells) obtained from naïve CD4^+^ T cells co-incubated with DCs pretreated with TGFβ_2_, IL-10, or DCs alone. The results are pooled data from four independent experiments (duplicate wells). Unstimulated DCs were used as a control. Right panels: representative plots of cytometric analysis of proliferating *de novo* induced Tregs after coculture with DCs treated with different cytokines. The data are expressed as the mean ± SEM (^#^*P* = 0.05, *P < 0.05, and **P < 0.01).

Bone marrow-derived DCs from HuBL mice were incubated in the presence or absence of TGFβ_2_ and pulsed with or without ApoB100 as described in the methods. The untreated DCs were characterized as CD11c^+^MHC-II^high^CD11b^+^DEC205^+^ (the detailed phenotype of DCs is shown in Figure S1 in Supplementary Material). Upon TGFβ_2_ treatment, the DCs exhibited a decreased surface expression of the co-stimulatory molecule CD86 and a modest reduction in the I-A^b^ MHC-II levels, compared with the controls (Figure 1A). Based on an analysis of the supernatants from these cells, we show that TGFβ_2_-induced tolerogenic DCs secrete lower levels of IL-12 and produce higher levels of IL-10 independently of being loaded with or without ApoB100 (Figures 1B–C). A trend toward reduced TNF secretion was observed in the TGFβ_2_-induced tolerogenic DCs (Figure 1D). Notably, TGFβ_2_-induced tolerogenic DCs presented also increased IDO1 protein expression (Figure S2 in Supplementary Material).

Next, we evaluated the capacity of the TGFβ_2_-induced tolerogenic DCs to induce the *de novo* expression of Foxp3 in naïve CD4^+^CD25^−^ T cells *in vitro*. Compared with the untreated or IL-10-treated DCs, the TGFβ_2_-treated DCs showed a superior capacity to induce Tregs (Figure 1E). Thus, TGFβ_2_-induced tolerogenic DCs were selected to be used as a tool to expand ApoB100-specific Tregs *in vivo*.

### Injection of TGFβ_2_-Treated ApoB100-Pulsed DCs Increases Treg Numbers in Atherosclerotic Plaques

**Figure 2:**
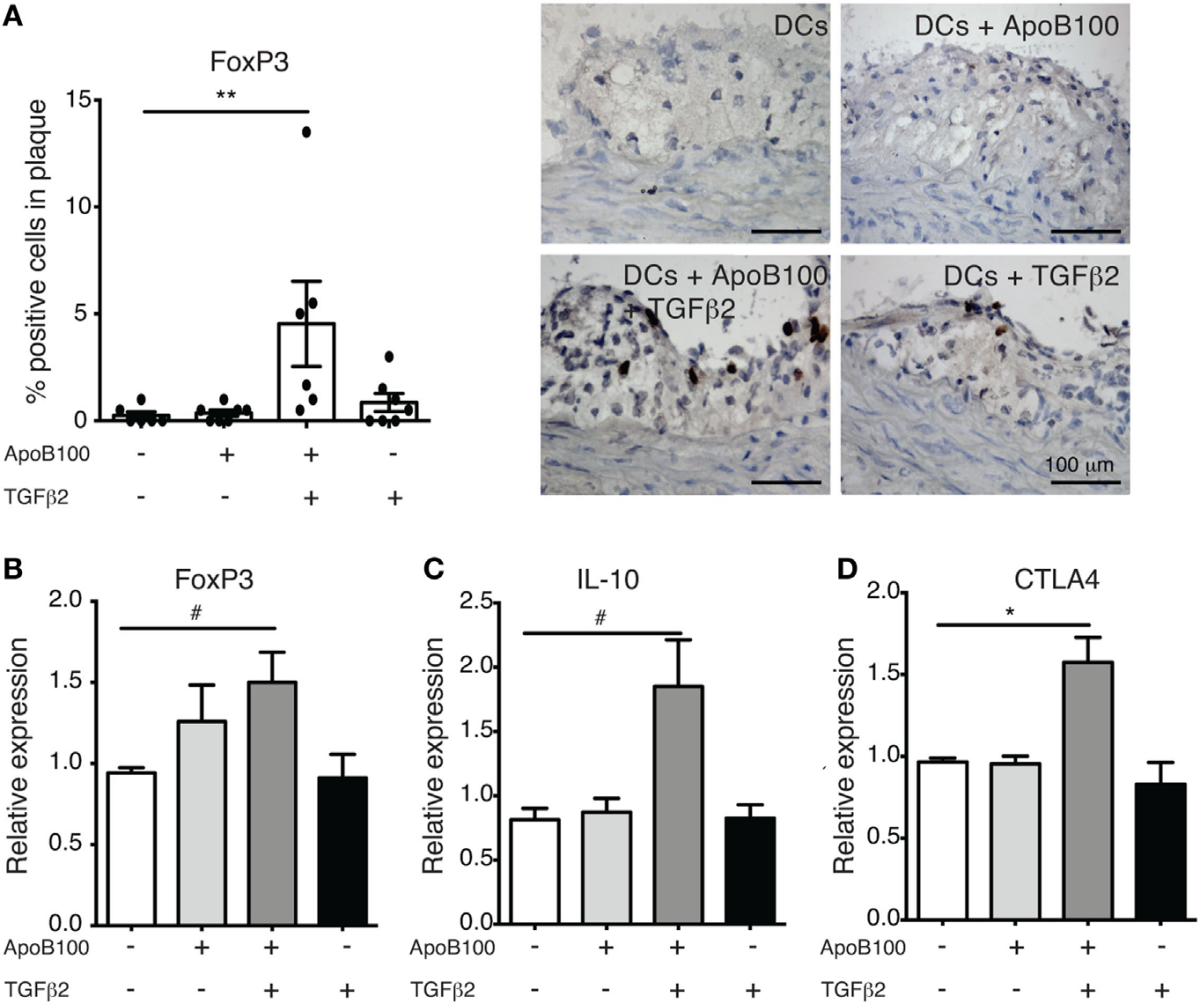
Tumor growth factor beta 2 (TGFβ_2_)-apolipoprotein B-100 (ApoB100)-loaded dendritic cells (DCs) increase regulatory T cell numbers in atherosclerotic plaques. **(A)** Analysis of immunostaining for FoxP3-positive cells in atherosclerotic plaques from mice injected with DCs alone (*n* = 6), DCs loaded with ApoB100 (*n* = 7), DCs loaded with ApoB100 and treated with TGFβ_2_ (*n* = 6), and DCs treated with TGFβ_2_ alone (*n* = 7). Right panels: representative microphotographs of staining of FOXP3-positive cells. **(B–D)** mRNA expression of FoxP3, interleukin 10 (IL-10), and cytotoxic T-lymphocyte associated protein-4 (CTLA4) in para-aortic lymph nodes from mice treated with different stimuli. The data are expressed as the mean ± SEM (^#^*P* = 0.05, *P < 0.05, and **P < 0.01).

HuBL mice were divided in four groups that received a single intravenous injection of (i) untreated, (ii) ApoB100-pulsed, (iii) TGFβ_2_-treated and ApoB100-pulsed, or (iv) TGFβ_2_-treated DCs. The immunohistochemistry analysis of the lesions revealed that only the TGFβ_2_-treated ApoB100-pulsed DCs substantially increased the FoxP3^+^ Treg numbers in the plaques (Figure 2A). Consistently, we observed an increased expression of Treg markers, including the mRNA levels of Foxp3, the anti-inflammatory cytokine IL-10, and the co-inhibitory molecule CTLA4, in para-aortic lymph nodes from the same group (Figure 2B–D).

### Treg Expansion Is Associated With Increased Arterial IDO1 Expression and Activity

**Figure 3:**
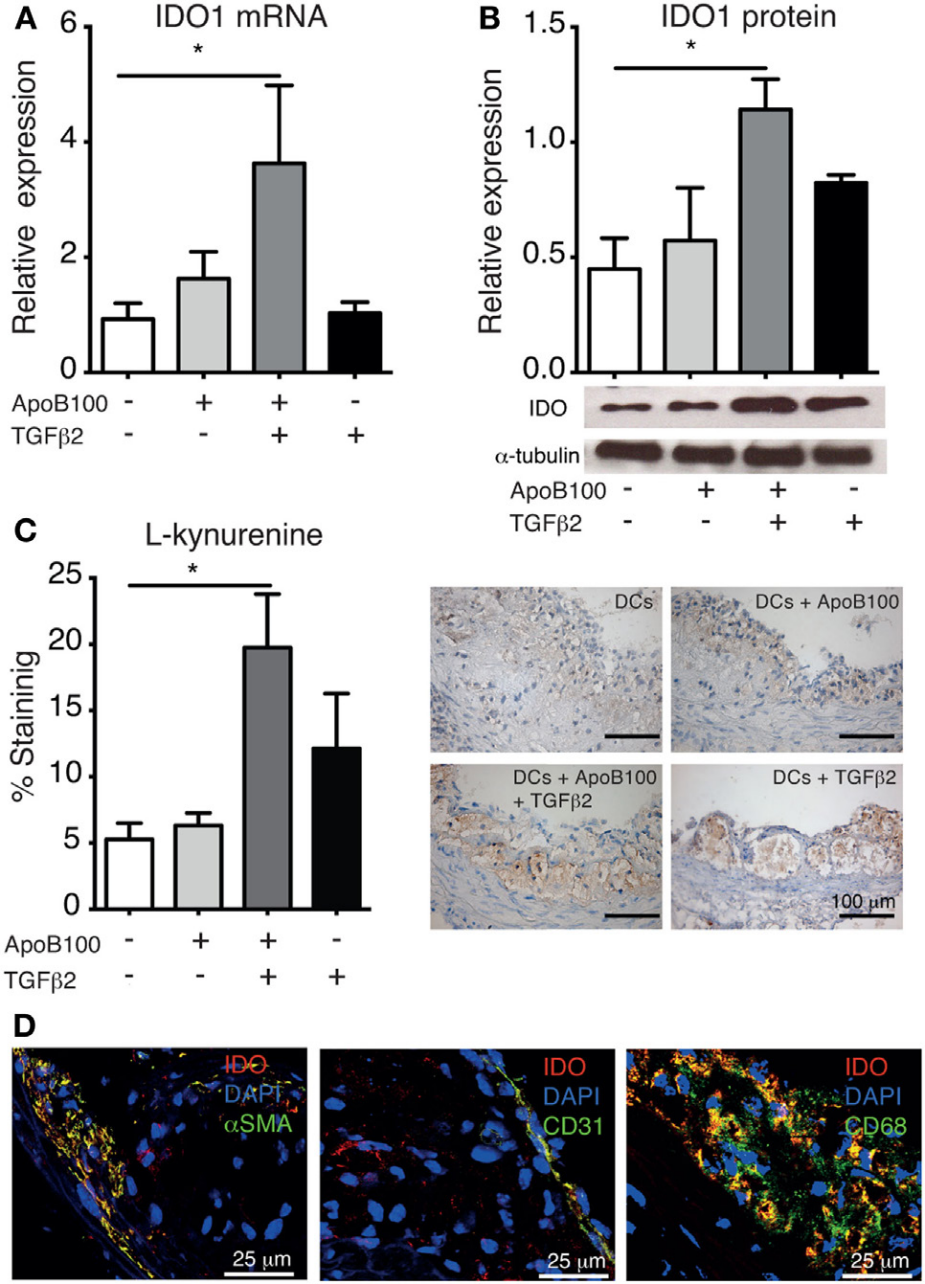
Tumor growth factor beta 2 (TGFβ_2_)-apolipoprotein B-100 (ApoB100)-loaded dendritic cells (DCs) increase arterial indoleamine 2,3-dioxygenase-1 (IDO1) expression. **(A)** IDO mRNA expression in abdominal aortas from mice treated with DCs alone (*n* = 6), DCs loaded with ApoB100 (*n* = 7), DCs loaded with ApoB100 and treated with TGFβ_2_ (*n* = 6), and DCs treated with TGFβ_2_ alone (*n* = 7). **(B)** Analysis of IDO1 protein expression in aortic roots. Right panel: representative immunoblot of IDO1 and alpha tubulin in aortic roots from mice treated with the different DCs. **(C)** Analysis of l-kynurenine-positive immunostaining in atherosclerotic plaques from different DC-treated mice. Right panels: representative microphotographs of l-kynurenine staining in atherosclerotic plaques from aortic roots. The data are expressed as the mean ± SEM (*P < 0.05). **(D)** Representative microphotographs of IDO co-localization (red) with SMA-positive smooth muscle cells (green, left panel), CD31-positive endothelial cells (green, middle panel), and CD68-positive macrophages (green, right panel).

Because increased antigen-specific Treg infiltration was observed in the vascular wall, we investigated whether IDO1 expression was concomitantly affected. Indeed, the Treg/IDO axis was triggered in the mice injected with TGFβ_2_-treated ApoB100-pulsed DCs. These mice exhibited increased arterial expression of IDO1 at the mRNA (Figure 3A) and protein levels (Figure 3B). In line with these data, mice receiving TGFβ_2_-treated ApoB100-pulsed DCs presented significantly increased l-kynurenine staining in their plaques (Figure 3C), suggesting a local increase in Trp metabolism in the vascular wall. A similar trend of increased IDO1 expression was observed in the para-aortic lymph nodes (Figure S3 in Supplementary Material).

Subsequently, we investigated IDO1 cell localization in the arteries in the same group. IDO1 co-localized with SMC (αSMA), endothelial cell (CD31), and macrophage (CD68) markers (Figure 3D). Whether the different DC-treatments systemically influenced IDO1 expression was first evaluated in mice spleens, but no differences were observed (Figure S3 in Supplementary Material). Consistently, no differences were observed in the plasma Kyn/Trp ratio among the groups (Figure S4 in Supplementary Material).

### Activation of the Treg/IDO Axis Reduces Atherosclerosis and Vascular Inflammation

**Figure 4:**
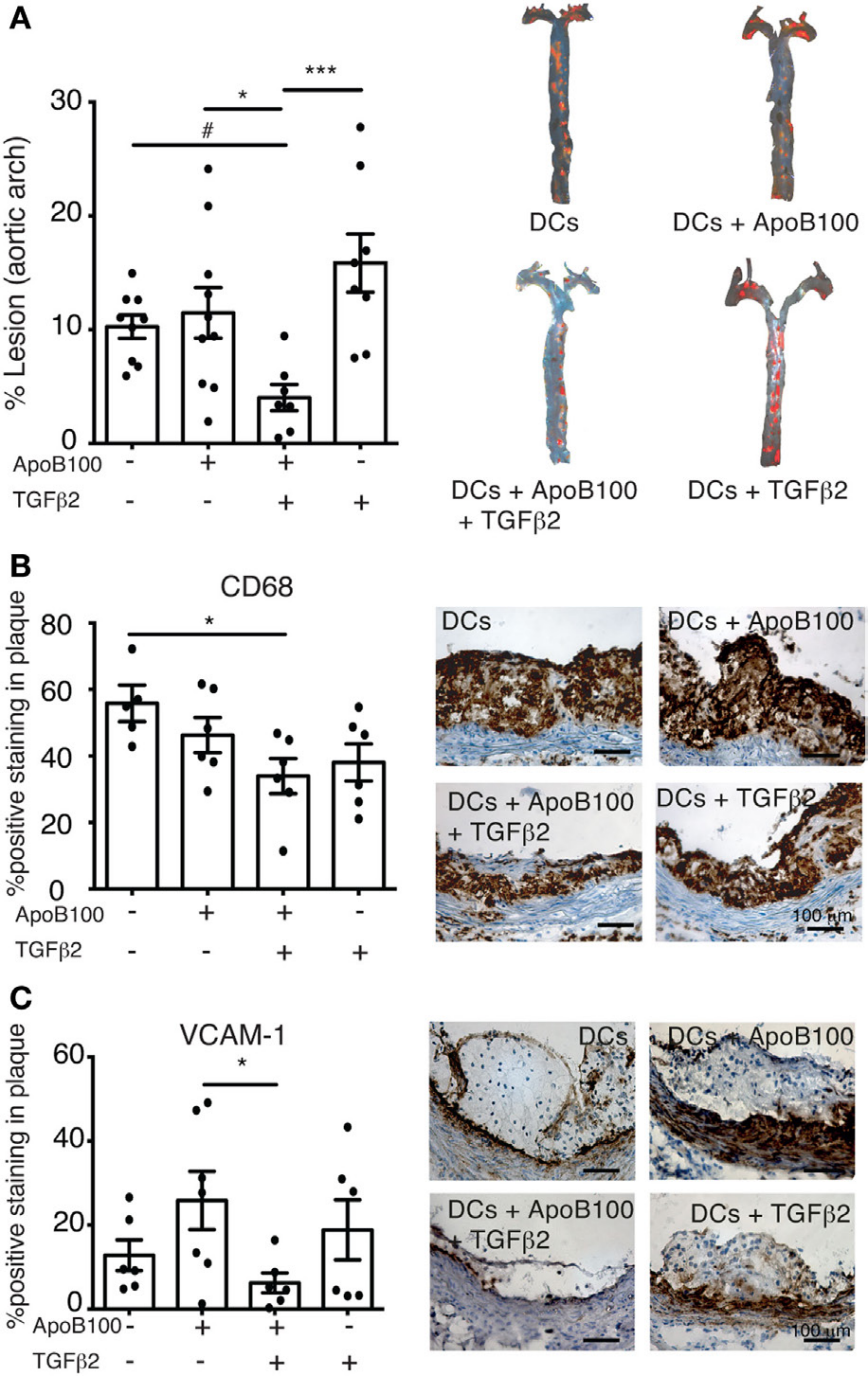
Activation of IDO–regulatory T cell axis reduces atherosclerosis lesions and vascular inflammation. **(A)** Quantitative analysis of atherosclerotic lesion areas in thoracic aortas from mice treated with dendritic cells (DCs) alone (*n* = 9), DCs loaded with apolipoprotein B-100 (ApoB100) (*n* = 10), DCs loaded with ApoB100 and treated with tumor growth factor beta 2 (TGFβ_2_) (*n* = 7), and DCs treated with TGFβ_2_ alone (*n* = 8). Right panels: representative images of mouse thoracic aortas showing Sudan IV plaque lipid staining. **(B)** Analysis of CD68-positive cell immunostaining in atherosclerotic plaques from different DC-treated mice. Right panels: representative microphotographs of CD68 immunostaining in atherosclerotic plaques from aortic roots. **(C)** Analysis of vascular cell adhesion molecule 1 (VCAM-1)-positive immunostaining in atherosclerotic plaques from DC-treated mice. Right panels: representative microphotographs of VCAM-1 immunostaining in atherosclerotic plaques from aortic roots. The data are expressed as the mean ± SEM (^#^*P* = 0.05, *P < 0.05, **P < 0.01, and ***P < 0.001).

We evaluated whether the Treg/IDO axis could influence plaque development in thoracic aortas from the four DC-treated groups. The mice receiving the TGFβ_2_-treated ApoB100-pulsed DCs exhibited a 75% decrease in the surface lesion area in the thoracic aorta compared with that in the other groups (Figure 4A). The reduction in the lesion area was associated with a significant reduction in plaque CD68 macrophage infiltration and VCAM-1 expression (Figures 4B–C). No differences in aortic M1 or M2 markers (Figure S5 in Supplementary Material), body weight and total plasma lipid levels were observed among the groups (Table 1).

**Table 1:** Body weight and lipid characteristics of the treated mice.

DC groups	*n*	Body weight (g)	Cholesterol (mg/dL)	Triglycerides (mg/dL)
Untreated	9	27 ± 1.0	1,099 ± 96	818.3 ± 94.2
ApoB100	7	29 ± 1.8	1,297 ± 124	1,087 ± 60.7
ApoB100 + TFGβ_2_	7	28 ± 1.2	1,338 ± 120	911.3 ± 134.0
TFGβ_2_	8	27 ± 0.6	1,687 ± 72	1,010 ± 189.7

*Data are expressed as the average ± SEM*.

*ApoB100, apolipoprotein B100; DC, dendritic cell; TGFβ_2_, tumor growth factor beta 2*.

### CTLA4-Ig Enhances IDO1 Expression and Activity in Activated Human Cultured Macrophages, SMCs, and Endothelial Cells

Cytotoxic T-lymphocyte associated protein-4 has been identified as a key effector molecule of Tregs (23). CTLA4 binding to CD80/CD86 has been shown to regulate cytokine-dependent IDO1 expression in DCs, leading to the mutual regulation of them and the T cells expressing CTLA4 (14). However, whether similar mechanisms operate in cells other than DCs have never been explored.

**Figure 5:**
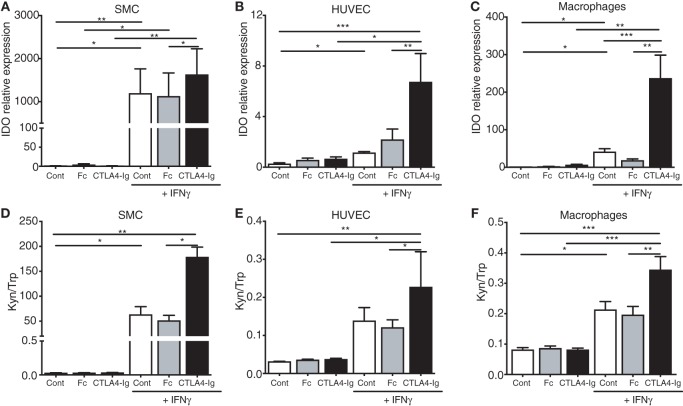
Cytotoxic T-lymphocyte associated protein-4 (CTLA4)-Ig enhances indoleamine 2,3-dioxygenase-1 mRNA expression and enzymatic activity in human smooth muscle cells (SMCs), endothelial cells, and macrophages. **(A–C)** IDO mRNA expression in human **(A)** SMCs, **(B)** human umbilical vein endothelial cells (HUVECs), and **(C)** macrophages cultured for 24 h with or without 400 U/mL IFNγ, washed and subsequently treated with CTLA4-Ig, isotype Fc, or medium alone. **(D–F)** Kynurenine to tryptophan ratio (Kyn/Trp) in the supernatants of human **(D)** SMCs, **(E)** HUVECs, and **(F)** macrophages cultured for 24 h with or without 400 U/mL IFNγ, washed and subsequently treated with CTLA4-Ig, Isotype Fc, or medium alone. The SMC results represent pooled data from five independent experiments (triplicate wells) using cells from two different donors. The HUVEC and macrophage results represent pooled data from five independent experiments each (triplicate wells) using cells from five different donors. Unstimulated cells were used as a control. The data are expressed as the mean ± SEM (^#^*P* = 0.05, *P < 0.05, **P < 0.01, and ***P < 0.001).

Expectedly, the IFNγ priming of human macrophages, SMCs, and endothelial cells upregulated IDO1 expression and activity *in vitro* (Figure 5; Figure S6 in Supplementary Material). A distinct fold change in IDO1 mRNA expression was observed among the different cell lines upon IFNγ stimulation (>1,000-fold in the SMCs; >30-fold in the macrophages; and 2-fold in the HUVECs). Interestingly, the concomitant treatment of these cells with CTLA4-Ig further increased the IDO mRNA levels (Figures 5A–C). Moreover, a significant increase in IDO activity was observed in the CTLA4-Ig-stimulated cells (Figures 5D–F), particularly in the SMCs. Notably, pre-stimulation with IFNγ led to increased CD80 mRNA levels on SMCs and HUVECs (Figure S7 in Supplementary Material), and no effects of CTLA4-Ig were observed in the absence of IFNγ simulation (Figure 5). CD86 mRNA was not influenced by treatments in macrophages and was undetectable in SMCs and HUVECs (Figure S7 in Supplementary Material).

## Discussion

In this study, we employed tolerogenic DC-based immunotherapy to induce Tregs specific to the atherosclerosis-related antigen ApoB100. This treatment led to an increased number of FoxP3^+^ Tregs and increased IDO1 expression in the vessel wall. Hence, the concomitant induction of Tregs and IDO1 was followed by a reduction in the atherosclerotic burden.

Tolerogenic DC-based immunotherapy could be a promising strategy to modulate atherosclerosis (5, 24). For example, we showed that IL-10 renders DCs a tolerogenic phenotype and the capacity to induce antigen-specific Tregs *in vivo* and prevent disease (5). While this approach remains at the experimental level in the cardiovascular field, tolerogenic DC-based clinical trials for the treatment of autoimmune diseases, including type 1 diabetes, rheumatoid arthritis (RA), multiple sclerosis, and Crohn’s disease, are underway (25).

In this study, tolerogenic DCs were used as a tool to investigate the relationship between Tregs and IDO in atherosclerosis. IDO1 is considered a major regulator of the immune system due to its ability to deplete Trp from the microenvironment, leading to the activation of general control non-derepressible 2 and the inhibition of basic cellular mechanisms, such as protein synthesis and immune cell division (26, 27). Equally important, the kynurenine pathway can lead to the generation of bioactive Trp metabolites, such as 3-hydroxyanthranilic acid (3-HAA), which can directly influence innate and adaptive immune cell inflammatory responses, including Treg induction (28).

Because TGFβ_2_ is highly expressed in immune-privileged sites and demonstrates potent immunosuppressive effects (29), we hypothesized that treating DCs with this cytokine could yield more Tregs *in vivo*. Indeed, the TGFβ_2_-induced tolerogenic DCs showed a robust capacity to induce Tregs *in vitro* and *in vivo*, suggesting that TGFβ_2_ may promote a more potent tolerogenic phenotype in DCs than IL-10 (5).

Regulatory T cells play an essential role in the maintenance of tolerance to self-antigens and suppressing excessive and deleterious immune responses. Similarly, in atherosclerosis, Tregs are associated with many protective functions, including the inhibition of effector T-cell responses (30), suppression of the maturation and immune-stimulatory capacity of DCs (31), modulation of macrophage- and endothelial cell-mediated pro-inflammatory responses (32, 33), and the promotion of proliferation and collagen production by SMCs (34).

While it is well known that Tregs can exert their immunosuppressive effects by secreting anti-inflammatory cytokines (3), a new mechanism has been recently described, the capacity to induce the immunomodulatory enzyme IDO (35). According to various studies, TGFβ or CTLA4 expression on Treg can regulate IDO in DCs and participate in the self-amplification of IDO1 expression and the maintenance of the tolerogenic phenotype of these cells (13, 36, 37). In humans, Treg activity is reduced in RA patients due to the epigenetically mediated downregulation of CTLA4, which leads to reduced IDO1 expression in APCs (14). Interestingly, tolerogenic DC-induced Treg suppression of collagen-induced arthritis was shown to be lost following pretreatment with the IDO inhibitor 1-MT (38).

Cytotoxic T-lymphocyte associated protein-4 is a co-inhibitory molecule constitutively expressed by Tregs that plays a critical role in peripheral tolerance (39). CTLA4 is homologous to CD28, which binds with a high affinity to CD80 or CD86 and contributes to the IDO-mediated regulatory T-cell generation through pathways that converge on non-canonical NF-κB signaling in APCs (40). While the CTLA4-mediated induction of IDO is well-established in the context of the immune synapse involving T cells and DCs (35), our finding showing that similar mechanisms regulate IDO1 in other cells in the vascular wall is completely novel. Moreover, our data suggest that CD80 rather than CD86 is the major transducer of CTLA4-mediated effects on vascular cells.

Our study shows that IDO1 is predominantly expressed in SMCs, macrophages and endothelial cells in the arterial wall. Expectedly, in our *in vitro* assays using these cells, IDO1 expression and Trp degradation increased upon IFNγ stimulation. Remarkably, CTLA4 substantially potentiated the IFNγ effects and increases IDO activity in these cells, particularly in the SMCs. These data are in line with previous studies indicating that IDO expression in vascular SMCs contributes to the enhancement of the natural resistance of the vascular wall to inflammation (41). In fact, we have previously shown that IDO-dependent Trp metabolism can influence the expression of VCAM-1 in human coronary artery SMCs *in vitro* and tunica media SMCs *in vivo* in *Apoe^−/−^* mice. Interestingly, in both cases, the VCAM-1 upregulation could be reversed by the administration of 3-HAA (11).

Our data suggest that Treg interactions with SMCs, macrophages and endothelial cells may not be restricted to soluble factors, e.g., TGFβ that regulate SMC proliferation and collagen production (42). Speculatively, the known role of IDO in the differentiation of Tregs (40) suggests that SMCs could play a role in Treg-induced “infectious tolerance” in the artery wall.

Although macrophages show a less pronounced increase in IDO activity upon CTLA4 ligation, they are the most abundant cell type in the plaque and their contribution to modulation could also be relevant. It has been shown that monocyte-derived macrophages can suppress T-cell proliferation *in vitro via* the rapid and selective degradation of Trp by IDO1 (43). Thus, the high rates of Trp degradation and the formation of bioactive Trp metabolites by SMCs, macrophages, and endothelial cells could constitute an important feedback loop that stabilizes and even enhances the effects of Tregs in the vessel wall.

The administration of the CTLA4-Ig fusion protein to hypercholesterolemic mice substantially reduces atherosclerosis, whereas a CTLA4 blockade accelerates disease (44). While it was not explored in the latter study, our data suggest that the IDO1 induction by CTLA4 could be an important atheroprotective mechanism induced by the treatment. Interestingly, CTLA4-Ig (abatacept) is currently under investigation as a first-line biologic for the treatment of autoimmune diseases, such as RA (45). Based on our findings, CTLA4-Ig emerges as a potential candidate for the prevention of CVDs due not only to its inhibitory effects on different immune cells but also to its IDO induction capabilities.

In conclusion, we show that the triggering of the “Treg–IDO axis” in the vascular wall generates a tolerance-sustained milieu characterized by reduced vascular inflammation and atherosclerotic lesion formation. Based on our study, strategies that reinforce the IDO1 pathway in a cell- or tissue-specific manner, such as the use of tolerogenic DCs and the induction of antigen-specific Tregs, have high therapeutic potential against atherosclerotic CVDs.

## Ethics Statement

All animal experiments were conducted according to the guidelines of Directive 2010/63/EU of the European Parliament on the protection of animals used for scientific purposes and approved by the Stockholm Norra regional ethical board.

## Author Contributions

DK and GH designed the project and supervised the research. MF, KP, RB, BS, MM, DJ, AH, and DK performed the experiments. MF, KP, RB, BS, and MM analyzed and interpreted data. GH contributed with the critical interpretation of data and revision of the manuscript. MF and DK wrote the manuscript.

## Conflict of Interest Statement

GH and AH hold patents on the use of tolerogenic DCs for the prevention and treatment of atherosclerosis. DK and GH hold patents on the use of 3-HAA for the prevention and treatment of hyperlipidemia and its complications. The remaining authors have no disclosures to report.
