# Supplementary material for: Activation of the Regulatory T-Cell/Indoleamine 2,3-Dioxygenase Axis Reduces Vascular Inflammation and Atherosclerosis in Hyperlipidemic Mice
Source: Front Immunol. 2018 May 7;9:950. doi: 10.3389/fimmu.2018.00950 (PMC5949314; doi:10.3389/fimmu.2018.00950)
Supplement: Supplementary file 1 [file Image_1.PDF]

SUPPLEMENTARY MATERIAL

Activation of the regulatory T-cell/Indoleamine 2,3-dioxygenase axis promotes vascular tolerance mechanisms and reduces atherosclerosis

Maria J. Forteza[1], Konstantinos A. Polyzos[1], Roland Baumgartner[1], Bianca E. Suur[2], Marion Mussacher[3], Daniel K. Johansson[1], Andreas Hermansson[1], Göran K. Hansson[1], Daniel F.J. Ketelhuth[1].

SUPPLEMENTAL FIGURES

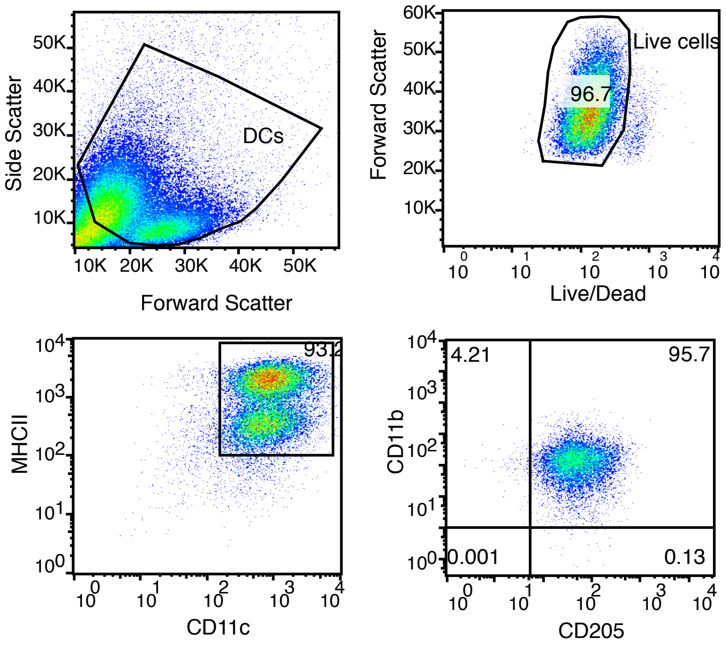

Supplementary figure 1: Gating strategy used for flow cytometry analysis of bone marrow-derived dendritic cells.

Bone marrow cells were differentiated into dendritic cells with IL-4 and GM-CSF, and purified by positive selection using CD11c magnetic cell sorting, as described in methods. Nonviable cells and cell couplets were gated out. Alive cells were evaluated for their expression of MHC-II IA^b, CD11b and CD205.
