# Supplementary material for: Activation of the Regulatory T-Cell/Indoleamine 2,3-Dioxygenase Axis Reduces Vascular Inflammation and Atherosclerosis in Hyperlipidemic Mice
Source: Front Immunol. 2018 May 7;9:950. doi: 10.3389/fimmu.2018.00950 (PMC5949314; doi:10.3389/fimmu.2018.00950)
Supplement: Supplementary file 2 [file Image_2.PDF]

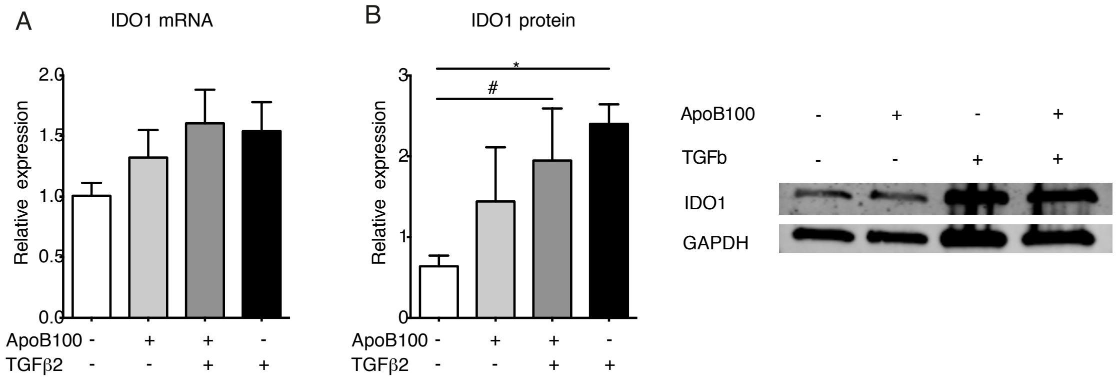

Supplementary figure 2: IDO1 mRNA and protein expression in bone marrow derived dendritic cells.

Quantitative analysis of IDO1 mRNA (A) and protein (B) from murine bone marrow derived dendritic cells untreated or treated with ApopB100 alone, TGFβ2 alone or treated with ApoB100 and TGFβ2 (n=3 for all groups). Values are expressed as mean ± SEM. #P=0.05, *P<0.05.
