# Supplementary material for: Activation of the Regulatory T-Cell/Indoleamine 2,3-Dioxygenase Axis Reduces Vascular Inflammation and Atherosclerosis in Hyperlipidemic Mice
Source: Front Immunol. 2018 May 7;9:950. doi: 10.3389/fimmu.2018.00950 (PMC5949314; doi:10.3389/fimmu.2018.00950)
Supplement: Supplementary file 3 [file Image_3.PDF]

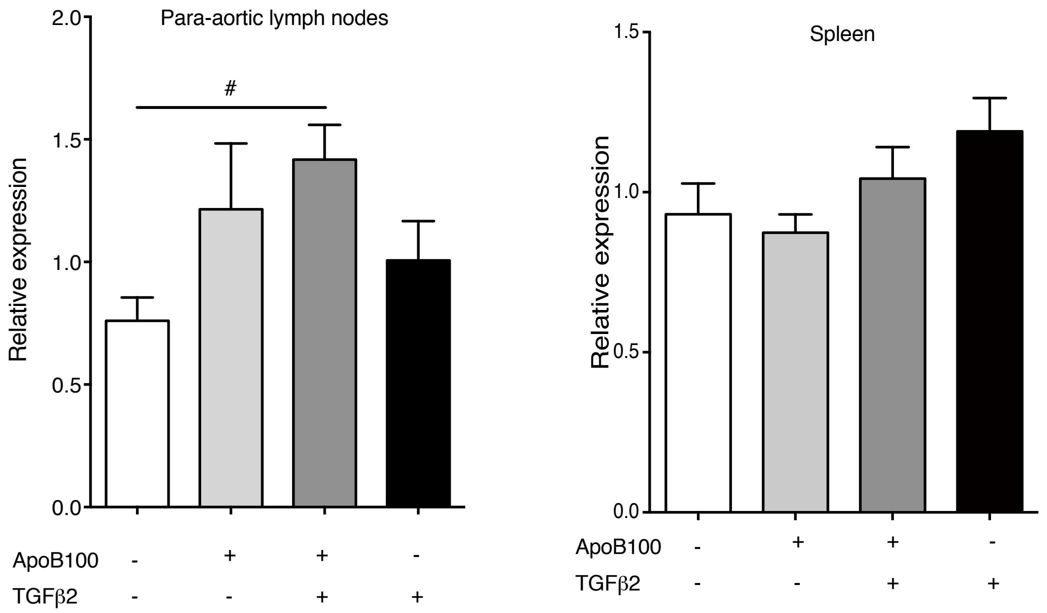

Supplementary figure 3: IDO1 mRNA in para-aortic lymph node and spleen samples.

Quantitative analysis of IDO in para-aortic lymph nodes (left panel) and spleen (right panel) from mice treated with DCs alone (n=9), DCs loaded with ApoB100 (n=10), ApoB100 and TGFβ2 (n=7), TGFβ2 alone (n=8). #) P=0.053.
