# Supplementary material for: Activation of the Regulatory T-Cell/Indoleamine 2,3-Dioxygenase Axis Reduces Vascular Inflammation and Atherosclerosis in Hyperlipidemic Mice
Source: Front Immunol. 2018 May 7;9:950. doi: 10.3389/fimmu.2018.00950 (PMC5949314; doi:10.3389/fimmu.2018.00950)
Supplement: Supplementary file 4 [file Image_4.PDF]

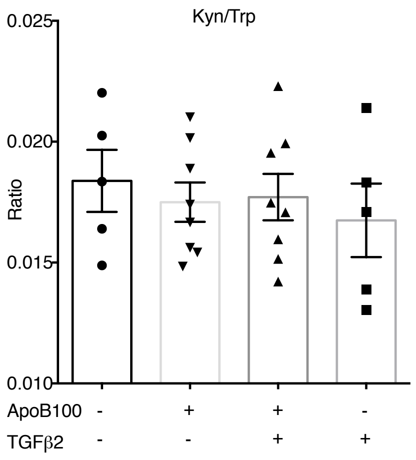

Supplementary figure 4: Evaluation of systemic IDO activity in plasma of DC-treated mice.

Analysis of the Kynurenine to Tryptophan ratio (Kyn/Trp) in plasma of mice injected with DCs alone (n=5), DCs loaded with ApoB100 (n=7), ApoB100 and TGFβ$_2$ (n=9), TGFβ$_2$ alone (n=5). Graphs show mean ± SEM of Kyn/Trp ratios.
