# Supplementary material for: Activation of the Regulatory T-Cell/Indoleamine 2,3-Dioxygenase Axis Reduces Vascular Inflammation and Atherosclerosis in Hyperlipidemic Mice
Source: Front Immunol. 2018 May 7;9:950. doi: 10.3389/fimmu.2018.00950 (PMC5949314; doi:10.3389/fimmu.2018.00950)
Supplement: Supplementary file 5 [file Image_5.PDF]

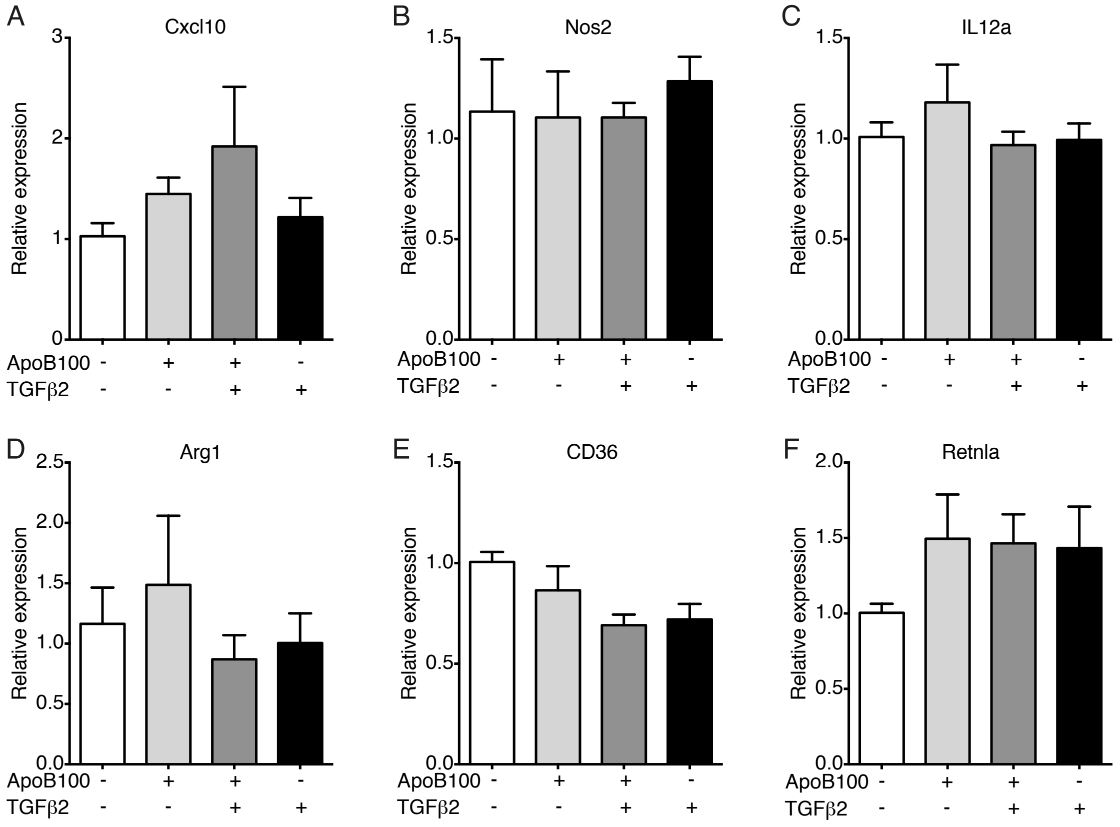

Supplementary figure 5: Analysis of M1 and M2 markers in aortas.

Quantitative analysis of mRNA for M1 macrophage markers: A) Cxcl10, B) Nos2, C) IL12a; and mRNA for M2 macrophage markers: D) Arg1, E) CD36, F) Retnla in aortas from mice treated with DCs alone (n=5), DCs loaded with ApoB100 (n=6), ApoB100 and TGFβ2 (n=4), TGFβ2 alone (n=8).
