# Supplementary material for: Activation of the Regulatory T-Cell/Indoleamine 2,3-Dioxygenase Axis Reduces Vascular Inflammation and Atherosclerosis in Hyperlipidemic Mice
Source: Front Immunol. 2018 May 7;9:950. doi: 10.3389/fimmu.2018.00950 (PMC5949314; doi:10.3389/fimmu.2018.00950)
Supplement: Supplementary file 6 [file Image_6.PDF]

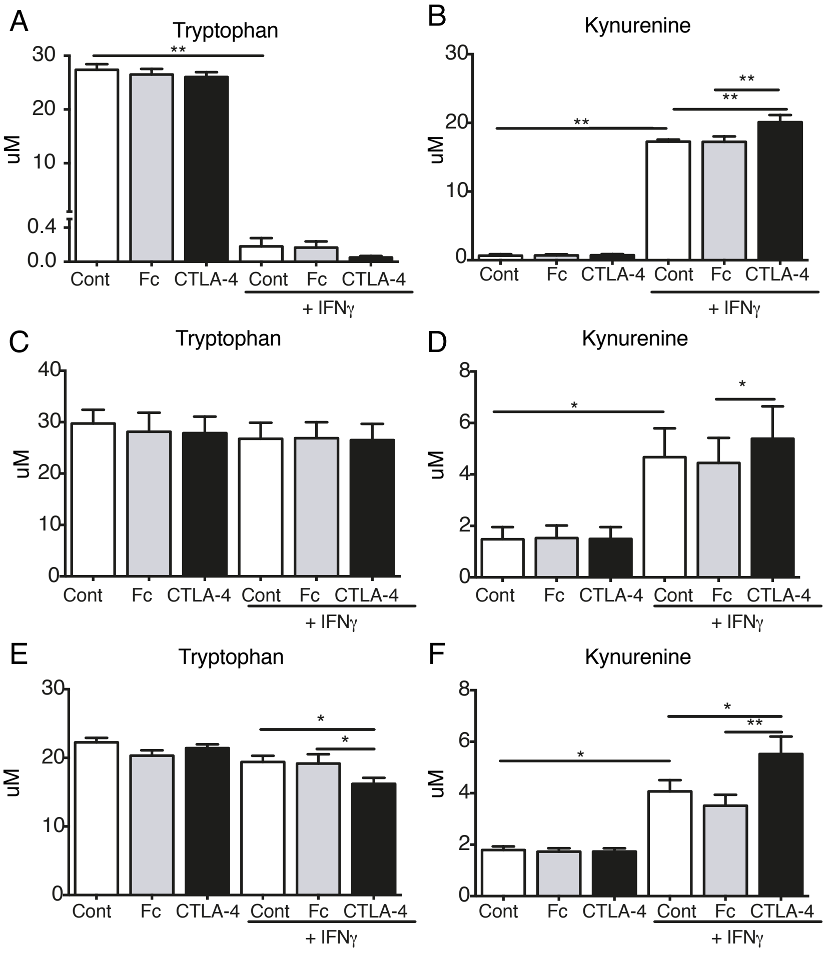

Supplementary figure 6: Quantitative analysis of kynurenine and tryptophan levels of supernatants of cultures of SMC, macrophages and HUVEC.

Left panels: Quantitative analysis of tryptophan in supernatants of (A) human SMC, (C) HUVEC and (E) macrophages pre-treated with or without IFNγ during 24h, washed and subsequently treated with CTLA4-Ig, Fc control or medium alone. **Right panels:** Quantitative analysis of kynurenine in supernatants of human (B) SMC, (D) HUVEC and (F) macrophages pre-treated with or without IFNγ during 24h, washed and subsequently treated with CTLA4-Ig, Fc control or medium alone. SMC results are pooled data from five independent experiments (triplicate wells) using cells from 2 pooled donors. HUVEC and macrophages results are pooled data from five independent experiments (triplicate wells) using cells from 5 pooled donors. IFNγ: Interferon gamma; HUVEC: Human umbilical cord endothelial cells; SMC: Smooth muscle cells. Values are expressed as mean ± SEM. #P=0.05, *$P<0.05$, **$P<0.01$.
