# Supplementary material for: Activation of the Regulatory T-Cell/Indoleamine 2,3-Dioxygenase Axis Reduces Vascular Inflammation and Atherosclerosis in Hyperlipidemic Mice
Source: Front Immunol. 2018 May 7;9:950. doi: 10.3389/fimmu.2018.00950 (PMC5949314; doi:10.3389/fimmu.2018.00950)
Supplement: Supplementary file 7 [file Image_7.PDF]

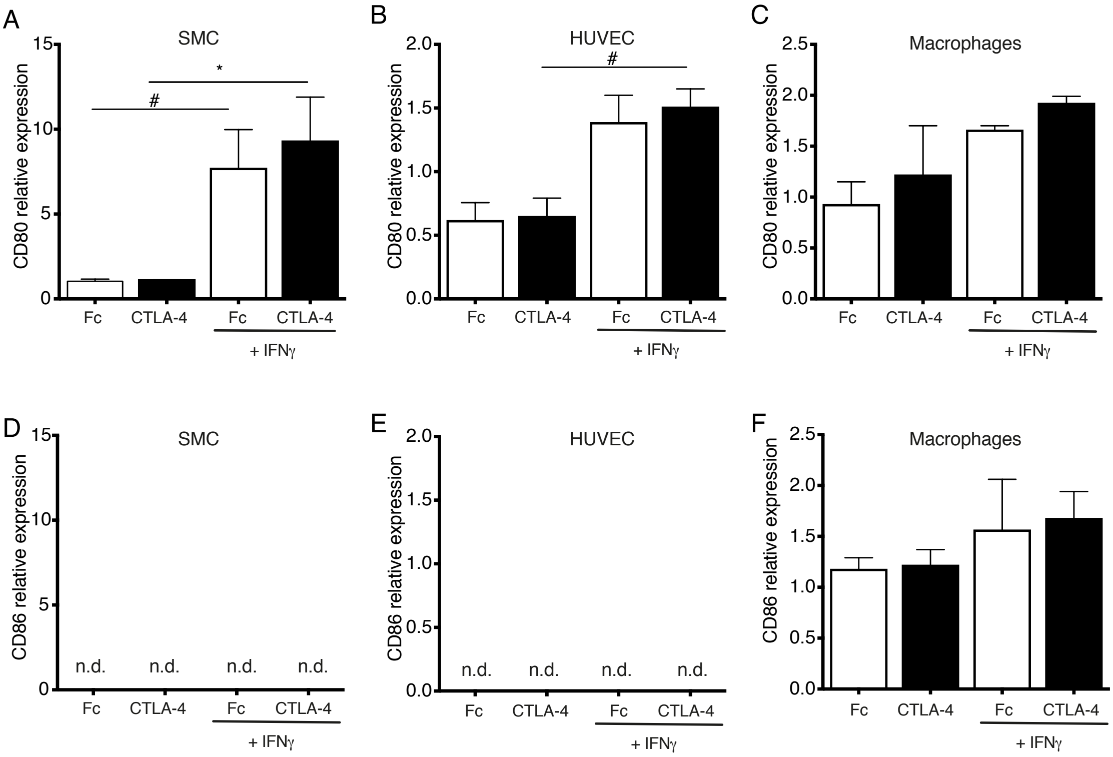

Supplementary figure 7: Analysis of CD80 and CD86 mRNA in cultures of SMC, macrophages and HUVEC.

Quantitative analysis of CD80 (B7.2) and CD86 (B7.1) in cultures of SMC (A, D), HUVEC (B, E) and macrophages (C, F) pre-treated with or without IFNγ during 24h, washed and subsequently treated with CTLA4-Ig, or Fc control. SMC results are pooled data from three independent experiments (triplicate wells) using cells from 2 pooled donors. HUVEC and macrophages results are pooled data from four independent experiments (triplicate wells) using cells from 4 pooled donors. IFNγ: Interferon gamma; HUVEC: Human umbilical cord endothelial cells; SMC: Smooth muscle cells. Values are expressed as mean ± SEM. #P=0.05, *P<0.05.
